# Clinical Management of Thrombotic Antiphospholipid Syndrome

**DOI:** 10.3390/jcm11030735

**Published:** 2022-01-29

**Authors:** Nor Rafeah Tumian, Beverley J. Hunt

**Affiliations:** 1 Haemostasis & Thrombosis Centre, Guy’s and St Thomas’ Hospital NHS Foundation Trust, Westminster Bridge Road, London SE1 7EH, UK; rafeah@ppukm.ukm.edu.my; 2 Clinical Haematology Unit, Department of Medicine, Faculty of Medicine, Universiti Kebangsaan Malaysia Medical Centre, Kuala Lumpur 56000, Malaysia

**Keywords:** antiphospholipid syndrome, autoimmune, acquired thrombophilia, thrombosis

## Abstract

Thrombotic manifestations of antiphospholipid syndrome are often a therapeutic dilemma and challenge. Despite our increasing knowledge of this relatively new disease, many issues remain widely unknown and controversial. In this review, we summarise the latest literature and guidelines on the management of thrombotic antiphospholipid syndrome. These include the laboratory assays involved in antiphospholipid antibodies (aPL) testing, the use of direct oral anticoagulants in secondary prevention, management of recurrent thrombosis, individuals with isolated aPL, and catastrophic antiphospholipid syndrome. Treatment aims to prevent the potentially fatal and often disabling complications of APS with antithrombotic and cardiovascular risks prevention strategies. Some insights and updates on topical issues in APS are provided. We also include our current practice, which we believe is the pragmatic approach based on the currently available evidence.

## 1. Introduction

Antiphospholipid syndrome (APS) is a rare, immune-mediated, acquired hypercoagulable disorder characterised by the presence of persistent antiphospholipid antibodies (aPL) in combination with clinical events of thrombosis in the venous, arterial, or microvascular system, and/or certain adverse pregnancy outcomes. Other common non-criteria APS manifestations include thrombocytopenia, livedo reticularis, or cardiac valve thickening or dysfunction [1,2]. The aPL that are included in the standard test panel are lupus anticoagulant (LA), anticardiolipin antibody (aCL), and anti-β2 glycoprotein-I antibodies (anti-β2GPI).

The diagnosis was initially based on criteria constructed at a 1999 meeting of experts at Sapporo [3], which was subsequently revised in 2006 in Sydney, and the latter is utilised today, as shown in Box 1 [4]. The terminologies of primary and secondary APS have been frequently used in the earlier literature. Primary APS is defined as APS occurring on its own, while secondary APS is when it occurs with other autoimmune conditions, for instance, systemic lupus erythematosus (SLE), autoimmune thyroid disorders, and coeliac disease [5]. However, overlapping autoimmune conditions may appear at any time, and the Euro-Phospholipid group reported that 36% had APS associated with co-presentation with SLE and 11% had APS associated with other diseases [2].

Limited data suggest that the global incidence of APS is approximately 5 cases per 100,000 persons per year, with an estimated prevalence of 40–50 cases per 100,000 persons [6,7]. The pathophysiology of APS remains not fully understood. Progress in our knowledge of APS has resulted in a more tailored approach to patient management. To date, the mainstay of treatment in APS is anticoagulation, which is mainly vitamin K antagonists (VKA) outside of pregnancy. Selection of the appropriate anticoagulant therapy should be based on the patient characteristics (age, underlying co-morbidities, bleeding risks, whether a woman is pregnant or contemplating pregnancy), the disease characteristics (type of vessels involved in thrombosis, site of thrombosis, type and number of aPL positivity, risk of recurrent thrombosis, and pregnancy loss), and treatment factor (type of anticoagulant available, safety and efficacy of anticoagulants, access to INR monitoring for VKA). The estimated 5- and 10-year overall survival from the Euro-Phospholipid Group was 95% and 91%, respectively [2,8].

Box 1 **The classification criteria for antiphospholipid syndrome based on the revised Sapporo criteria [4].**

**Clinical criteria**

Vascular thrombosis

≥1 clinical episode of arterial, venous, or small-vessel thrombosis. Thrombosis must be confirmed by objective validated criteria (i.e., unequivocal findings of appropriate imaging studies or histopathology). If histopathological confirmation is used, thrombosis must be present without evidence of inflammation of the vessel wall.

Pregnancy morbidity

- ≥1 unexplained death of a morphologically normal foetus at or beyond the 10th week of gestation, or
- ≥1 premature delivery of a morphologically normal foetus <34 weeks gestation due to severe pre-eclampsia or eclampsia (defined according to standard definitions) or recognised features of placental insufficiency, or
- ≥3 unexplained recurrent miscarriages at <10 weeks of gestation, with the exclusion of maternal and paternal factors (such as anatomical, hormonal, or chromosomal abnormalities)

**Laboratory criteria**

The presence of antiphospholipid antibodies on ≥2 occasions at least 12 weeks apart and <5 years before clinical manifestations, as demonstrated by ≥1 of the following:

- Presence of lupus anticoagulant in plasma
- Medium to high titre of anticardiolipin antibodies (>40 GPL * or MPL *, or >99th percentile ^$^) of immunoglobulin G (IgG) or IgM isotypes in serum or plasma
- Anti-β2-glycoprotein 1 antibodies of IgG or IgM isotypes present in serum or plasma

Note: * GPL and MPL are arbitrary units; 1 GPL or MPL represent 1 μg/mL of affinity-purified IgG or IgM antibody, respectively; ^$^ The exact value depends on the assay.

## 2. Antiphospholipid Antibodies

To date, the exact cause or trigger of the production of aPL by B lymphocytes is uncertain. Current hypotheses are that there is an exposure of cryptic epitopes on β2-glycoprotein I during bacterial infection, which, in some individuals, leads to the production of autoantibodies to this cryptic epitope [9,10]. Genetic studies using different methods, such as the human leukocyte antigen (HLA) system investigating the HLA-DR markers [11,12] and identification of potential susceptible gene/loci and lead single-nucleotide polymorphisms (SNPs) for production of aPL using whole-exome sequencing [13] and genome-wide association studies [14], showed a genetic susceptibility in patients and family members with APS as in other autoimmune diseases. It is important to remember that presence of aPL does not imply that the patients will necessarily develop the full syndrome.

### Antiphospholipid Antibody Testing

All three tests, LA, aCL, and anti-β2GPI, should be performed twice, at least 12 weeks apart to confirm the diagnosis of antiphospholipid syndrome (APS) [4]. Testing for all three aPL ensures that those who may be single positive for one antibody are not missed and repeated testing avoids overdiagnosis as the transient presence of aPL is common.

The important indications for testing include unprovoked thrombosis at common sites, such as deep vein thrombosis (DVT) and pulmonary emboli (PE) and unusual sites, such as cerebral venous sinus or splanchnic vein thromboses; recurrent thrombosis in patients receiving anticoagulation that is unexplained by subtherapeutic anticoagulation, poor adherence, or cancer-associated thrombosis; young patients with an arterial thrombotic event; patients having a combination of arterial and venous thrombi; those with unexplained sudden onset of multiorgan failure due to small vessels ischaemia, who do not have disseminated intravascular coagulation; and patients with pregnancy morbidity such as recurrent first trimester miscarriages, as outlined in international guidelines and publications [15,16].

Antiphospholipid antibodies are heterogeneous in nature; therefore, there is currently no specific test that can detect all of them [16]. Detection of LA is based on their in vitro effect on functional phospholipid-dependent coagulation assays. Intra- and inter-laboratory inconsistencies exist in LA testing rendering it difficult to standardise LA detection worldwide. International guidelines have recommended that two different functional assays need to be performed in a three-step procedure (screen-mix-confirm) [15]. Dilute Russell viper venom test (dRVVT) is the first choice method of testing for LA [15].

The principle of the LA assay is that aPL will bind to the phospholipid in the assays and, thus, cause a prolonged phospholipid dependent coagulation time due to the antibody, preventing binding of the coagulation factors to phospholipid where they interact. Secondly, the 1:1 ratio of patient: pooled normal platelet-poor plasma immediate mixing test will not usually correct the coagulation time if aPL is present. For the most important part of the assay, the LA confirmation, a large amount of phospholipid is added to neutralize the activity of aPL; thus, the coagulation time will be normalised. There are currently several LA assays that inevitably contribute to the inter-assay differences with regards to the preparation of sample, reagents, and methods performed in the studies and interpretation of results. Table 1 illustrates several tests being performed to detect LA.

The timing of blood sampling with the onset of thrombosis and presence of active infection is also important given that high factor VIII levels in the acute phase will shorten the aPTT results and can cause false-negative results whilst elevated C-reactive protein can cause false-positive results as was demonstrated in the COVID-19 pandemic [15]. The information on patients’ anticoagulation therapy is important as this may influence the results and interpretation of the tests.

In our centre, dilute Russell viper venom test (dRVVT) and dilute activated partial thromboplastin time (aPTT) are both performed. If the patients are receiving oral anticoagulation, except for dabigatran, the Taipan snake venom test (TSVT) [17] (see Table 1 for explanation) will be added to the tests. The final results of the LA tests should be reported as positive or negative. As opposed to LA tests, aCL and anti-β2GPI testing are performed using solid-assay and are most commonly detected by enzyme-linked immunosorbent assay (ELISA) or other detection systems, such as chemiluminescent techniques [18]. The aCL and anti-β2GPI testing are not affected by anticoagulants [18].

## 3. Management of Patients with Thrombotic APS

APS is usually not a ‘full house’ syndrome, in which women with aPL will not necessarily be affected in pregnancy, although they may have thrombotic events or visa-versa [19]. A minority of patients may have both. In general, reducing thrombotic risk by managing cardiovascular risk factors, namely obesity, diabetes mellitus, hypertension, hyperlipidaemia, and smoking through lifestyle modifications, regular exercise, and medical therapies, despite the absence of clinical trials showing their benefits, is still considered to be important in managing all patients with aPL and APS.

Unlike the inherited thrombophilias, which cause venous thromboembolism, in APS, any vessel can be subjected to thrombosis; thus, the presenting signs and symptoms can vary enormously depending on the vessel affected and with different frequencies of occurrence. Population-based and cohort studies from the USA, Italy, Europe, Japan, Korea, and Singapore have collectively shown a variable trend in the prevalence of arterial and venous thrombotic events in APS patients [1,2,6,20,21,22,23]. The disparities could be explained by the methodological differences that further contributed to selection bias, variations in the environmental and possibly genetic factors across geographical regions, or may reflect differences in referral patterns to the tertiary level hospitals with specialised clinics. For example, we tend to see many newly diagnosed APS patients presenting with DVT and PE in our haematology clinics and yet, our lupus clinics have many patients who presented with cerebral events.

- Pathophysiology and risks

The pathophysiology of thrombosis in APS is not fully elucidated. Antiphospholipid antibodies, particularly anti-β2GPI, will bind to domain 1 of β2GPI if β2GPI is bound to a negatively charged surface. Negatively charged surface phospholipid surfaces occur due to the flip-flopping of bilipid membranes during activation or damage of endothelial cells, monocytes, and platelets [24,25]. A ‘second-hit’ process involving the interplay between complement activation and coagulation cascade may further exaggerate thrombus formation [26,27].

The risks of thrombosis are loosely associated with the types and titres of aPL. The presence of all three, LA, IgG and/or IgM anti-β2GP,I and IgG and/or IgM aCL positivity, known as ‘triple aPL-positivity,’ is particularly associated with a high risk of thrombosis based on the cumulative incidence of thromboembolic events of 12% (95% CI, 10–15), 26% (95% CI, 22–30), and 44% (95% CI, 39–50) after 1, 5, and 10 years, respectively [28].

- Thrombosis prediction tool

A risk assessment model to predict thrombotic risk has been developed. The Global APS Score (GAPSS) was developed based on the calculation of thrombosis risk using aPL profile and particularly cardiovascular disease risk factors [29]. It was validated in patients with SLE. The latter Adjusted Global Anti-Phospholipid Syndrome Score (aGAPSS) excluded antiphosphatidylserine/prothrombin antibodies (aPS/PT) from the previous GAPSS scoring algorithm given these antibodies are not currently included in the criteria for defining APS and not all laboratories routinely test for aPS/PT [30]. Using the APS patients with a history of documented thrombosis from the AntiPhospholipid Syndrome Alliance For Clinical Trials and International Networking (APS ACTION) Clinical Database and Repository, a cross-sectional study showed that, in patients with recurrent thrombosis, those with recurrent arterial as compared to venous thrombosis had higher aGAPSS [31].

### 3.1. Acute Thrombosis

DVT and PE constitute the most common venous thromboembolism (VTE) events, with the frequencies of 40% and 14%, respectively, in the Euro-Phospholipid Project Group [2]. Meanwhile, the most common arterial thrombotic manifestations are cerebrovascular accidents, either stroke (20%) or transient ischemic attacks (11%), and thrombotic myocardial infarction (5.5%) [2]. In a critical review by the APS ACTION group, there were 10%, 11%, and 14% of aPL positivity in general-population patients with DVT, myocardial infarction (MI), and stroke, respectively [32]. For example, a recent cross-sectional single-centre study reported that the prevalence of APS was 9% among 18–50 years old patients with first-episode unprovoked VTE [33]. Six out of the 44 APS patients also had SLE [33]. Thus, APS is not uncommon in patients with unprovoked VTE and physicians should test for aPL in those presenting with unprovoked VTE, especially where there is uncertainty about long term management.

Patients with a first episode of acute VTE should be treated according to the standard international guidelines using either DOACs from the start or the choice of low molecular weight or unfractionated heparin, followed by DOACs or vitamin K antagonist, depending on the available resources, for a minimum of three to six months of anticoagulation [34,35]. Clinicians should assess all VTE cases for underlying transient provoking factors such as the use of combined oral contraceptive pills, pregnancy, recent immobilisation, history of recent surgery, or acute medical illness. After completion of the primary treatment, APS patients with provoked VTE may stop anticoagulant therapy despite the presence of persistent aPL, but should be offered a follow up.

In the event of acute arterial thromboses, patients will be managed as per standard management of ischaemic stroke, acute ST-elevation myocardial infarction (STEMI), non-STEMI, or peripheral limb occlusion. The clinical approach depends on the site of the thrombosis, patients’ haemodynamic status, and availability of the hospital facilities with clinical expertise during the presentation. They should be aggressively treated with thrombolytic therapies or reperfusion strategies as per clinical need, like any other patients. For instance, patients with acute MI will initially be treated medically with thrombolytic therapy or primary percutaneous coronary intervention, and subsequently treated with antiplatelet therapy. However, the primary pathology in APS-related acute arterial events is aPL-related thrombus formation rather than atherothrombosis, especially in patients without cardiovascular risk factors. A systematic review showed that some of the features of patients with APS-related thrombotic MI include younger than the typical AMI patients, an increased percentage of female patients, and lower platelet counts at presentation [36]. The hallmark of a thrombotic MI is the absence of atherosclerosis on coronary angiogram with normal coronary arteries under the thrombosis [36]. Although these differentiating features may not have any major implications during the acute management of thrombosis, we argue that a different treatment approach is required in the long-term management, in which patients with thrombotic MI due to APS should have long term VKAs rather than usual MI post-event care. The optimal therapeutic approach of acute arterial events in patients with APS continues to be debatable and more prospective studies are required.

### 3.2. Secondary Prevention

The aim of long-term management in APS is the prevention of recurrent thrombosis without excess side effects from anticoagulation. Among the earliest study assessing the risk of recurrent thrombotic events among APS patients with unprovoked VTE, Khamashta et al. reported that 69% of 147 patients had recurrent thrombosis with the median time between the initial thrombosis and the first recurrence of 12 months [37]. The rate of recurrent thrombosis was highest (1.3 per patient-year) during the first six months following the cessation of warfarin therapy [37].

Given the evidence that APS patients with arterial thromboses and unprovoked VTE require indefinite anticoagulants, the other important issues to be addressed are the optimal intensity and ultimate duration of anticoagulation, and more recently, the role of DOACs. Systematic reviews that included two important randomised trials [38,39] on different INR targets have shown that higher intensity of VKA (warfarin with a target INR of 3.1 to 4.0) versus standard-dose VKA (warfarin with a target INR of 2.0 to 3.0) did not show any differences in the rates of thrombotic events [40,41]. Nevertheless, there were limitations to the study in that the INR in the high-intensity group was below the target range 43% of the time and those with previous recurrent events on anticoagulation were excluded. The INR ranged between 2.0–3.1 during 86% of the time that it was subtherapeutic [38]. The rate of major bleeding was comparable between the two randomised trials [38,39]. However, the WAPS study reported an increased risk of minor bleeding in the high-intensity warfarin group (HR 2.9, 95%CI, 1.1–7.5) during a mean of 3.4 years of follow-up [39].

Following the approval of Direct Oral AntiCoagulants (DOACs) by the US Food and Drug Administration (FDA) in 2010 for the treatment of VTE, multiple studies were performed to investigate the efficacy and safety of DOACs in the secondary prevention of thrombotic APS. The advantages of DOACs over vitamin K antagonists include ease in administration and lack of a need to monitor. In a systematic review and meta-analysis of seven studies including 835 patients, those on DOACs (mostly on rivaroxaban) had a significantly higher risk of thromboembolic events as compared to VKA (risk ratio (RR) 1.7, 95% CI 1.1 to 2.6) [40]. In studies assessing rivaroxaban only, the risk was about thrice higher (RR 3.4, 95% CI 1.5 to 7.4) [40]. A 2020 Cochrane report demonstrated that those with APS receiving rivaroxaban had an increased risk of stroke (RR 14.1, 95% CI 1.9 to 106.8), but there was no significant differences in any thromboembolic event including deaths [41]. The risks of major bleeding and all bleeding events were not significantly different in both reviews [40,41].

These systematic reviews led to current international guidelines for not recommending DOACs for secondary prevention of thrombotic APS, especially in the context of arterial thrombosis and triple positive aPL patients [42,43]. Very recently, a prospective randomised trial study reported a higher percentage of stroke in the apixaban group of thrombotic APS patients compared to warfarin, using a lower dose of apixaban at 2.5 mg twice a day and, thus, it was terminated early [44].

Our recent retrospective study on DOACs in 82 APS patients showed a similar recurrence rate with previous studies on APS with VTE and 1.2% of recurrent stroke [45,46,47]. Therefore, in our current practice, patients with triple-positive thrombotic APS are treated with VKA with target INR 2–3 or INR 3–4 (in arterial thrombosis) and with consideration of the individual’s risk of bleeding and recurrent thrombosis, in line with the current international recommendations [42,43] (Table 2). A prospective observational study evaluating the long-term outcomes of non-high-risk APS patients on DOACs is ongoing and may change our approach in the near future [48].

### 3.3. Recurrent Thrombosis While on Treatment

Despite VKA therapy, the rate of recurrent thrombotic APS remains high, ranging between 3 to 24% within 5 years [2,38,39]. The variable rates could be explained by the difficult APS cases seen in the tertiary referral centres and poor INR control and treatment adherence. The occurrence of recurrent thrombosis while on anticoagulants is a challenging situation to manage. Confirmatory radiological imaging is mandatory to ascertain the development of new thrombosis or propagation of the existing thromboembolic event. It is important to ensure adequate control of cardiovascular risk factors, non-compliance to treatment, and INR control if taking a VKA, any possible drug-drug, or drug-food interactions. In addition, a history, complete physical examination, and biochemical tests, such as serum paraprotein and light chain assay, should be performed to exclude solid organ or haematological malignancies. Our recent study showed that some APS patients with recurrent thrombosis despite adequate anticoagulation had a paraprotein [49]. Doyle et al. showed that the percentage of other autoimmune disorders among these nine patients was low and there was a predilection for arterial thrombosis [49]. The rate of recurrent thrombosis was significantly higher in those with monoclonal gammopathy while on anticoagulation [49].

The present management of recurrent thrombosis while receiving standard-intensity VKA with the target INR range of 2.0–3.0 is to increase VKA to a high-intensity regimen, with a target INR of 3.5 (range 3.0–4.0), following an interim bridging with supplementary LMWH [50,51]. It is recommended that these patients should be on indefinite warfarin with a target INR of 3.0 to 4.0 and close INR monitoring [42,50,51]. Some physicians add aspirin with a moderate warfarin goal of a target INR of 2.0 to 3.0, but we do not practise this in our centre given the increased risk of bleeding.

Fondaparinux, a synthetic pentasaccharide with anti-Xa activity, may have a beneficial role in patients with recurrent thrombotic events while receiving other anticoagulation. Although it has not been formally investigated in APS, fondaparinux has been widely used as an alternative to LMWH in patients who have an allergy or are intolerant to LMWH and also in the setting of heparin-induced thrombocytopenia (HIT) [52,53]. Other benefits of fondaparinux are that it is not associated with HIT or osteoporosis.

Adjunctive therapy such as hydroxychloroquine (HCQ), an immunomodulatory agent, and statins, lipid-lowering agents, both of which have antithrombotic effects, may be considered in refractory APS [54,55,56,57].

### 3.4. Isolated aPL

For asymptomatic individuals with isolated aPL, the natural clinical course of the disease is not well known. Therefore, this has caused a dilemma to the treating physicians on whether this group of individuals would benefit from primary thromboprophylaxis. Several retrospective and prospective studies were conducted to investigate the risks of developing thrombotic events among those with aPL positive. The thrombosis rates were reported between 0.8 to 5.3 per 100 patient-year [58,59,60,61,62] with the pooled random effect estimate of 1.7 thromboses per 100 patient-year (95% CI 1.1–2.2) [61]. The results were highly variable given that the study subjects were heterogeneous in terms of aPL positivity, presence of SLE or autoimmune disease, and other risk factors for venous and arterial thrombosis. A recent study showed that triple-positive aPL had a four-fold higher risk than single positive [61]. Male sex [58], smoking [60,61], hypertension [59], and triple positive aPL [61] were among the independent risk factors for thrombosis.

Given the risk of thrombosis among aPL positive individuals, the need for thromboprophylaxis has become a matter of debate. In an earlier randomised clinical trial, the Antiphospholipid Antibody Acetylsalicylic Acid (APLASA) study was unable to show a protective role against thrombosis [62]. There are several potential reasons that may have contributed to the findings. The final number of patients recruited was not powered to test the effect of low dose aspirin (LDA), not all research patients reported adherence to the study medication, 12% reported significant interruption of study, and there was a short study follow-up [62]. In a meta-analysis by Arnaud et al. involving 11 studies and a total of 1208 patients, LDA reduced the risk of a first thrombotic event among asymptomatic aPL individuals, patients with SLE, or obstetrical APS [63]. However, the majority of the studies in the meta-analysis were observational.

The 2019 EULAR Guidelines recommended the use of LDA for primary thromboprophylaxis in asymptomatic aPL individuals with high-risk profiles regardless of the cardiovascular risk factors [42]. The high-risk profile group includes the presence of LA as it is most related to thrombosis, the presence of double (any combination of LA, aCL, or anti-β2GPI) or triple (all three subtypes) aPL positivity, or the presence of persistently high aPL titres [42].

However, in our practice, we currently do not use aspirin as primary prevention in healthy patients with aPL given the current evidence is not strong and the risk of aspirin-induced bleeding has recently been identified to be substantially greater than the antithrombotic benefits. The evidence was observed in three pivotal trials involving moderate-risk patients (ARRIVE study) [64], diabetic patients (ASCEND study) [65], and elderly people over 70 years (ASPREE study) [66]. We offer regular follow-ups in hospital- or community-based clinics regularly with a strong advocate of maintaining a healthy lifestyle and prevention/reduction of obesity. If they undergo surgery or have an acute medical illness, thromboprophylaxis is given [67]. Certain medications such as combined oral contraceptive pills and oral hormone replacement therapy (HRT) are avoided, but the progestogen-only pills, the MIRENA coil or transdermal HRT are safe.

### 3.5. Special Situation: Cerebral APS

Involvement of the brain in APS (cerebral APS) deserves special attention as the spectrum of neurological manifestation varies widely given that cerebral thrombosis can occur at any level within the vasculature of the brain. The site and extent of thrombotic involvement determine the type of neurological deficits. Stroke and TIA are the most common manifestations, being the initial presentation in almost 30% of adults with APS [2], and they involve cerebral arteries. Cerebral venous sinus thrombosis (CVST), as a venous thrombus, has a prevalence of 0.7% [68], and most commonly presents as a headache. Apart from lacunar infarcts, medium-sized vessel thrombosis can involve the central retinal artery and vein, causing acute visual acuity loss and visual field defect, respectively. Another unique feature in cerebral APS is the involvement of small vessels or microvascular thrombosis. Depending on the site of microvascular thrombosis, the neurological manifestations widely vary, in which cognitive impairment usually occurs. This is often associated with periventricular lesions [69]. Movement disorders such as chorea and hemiballismus have been seen occasionally [70]. There is some evidence suggesting non-thrombotic related brain changes that are related to immune-mediated vascular, inflammatory, and direct neuronal injury by aPL [69,71].

Neuroimaging, especially magnetic resonance imaging, is crucial to delineate the underlying site and extent of central nervous system involvement. In our practice, the mainstay therapy of secondary prevention in cerebral APS remains as VKA with a target INR of 3.0–4.0. However, in elderly patients, we tend to reduce the target INR to 2.0–3.0 provided patients do not develop new symptoms and there are no new changes in neuroimaging.

### 3.6. Catastrophic Antiphospholipid Syndrome

Catastrophic antiphospholipid syndrome (CAPS) is an acute, life-threatening yet rare manifestation of APS involving multiple vascular occlusions, usually small vessels, resulting in multiorgan failure in the presence of high circulating aPL. The criteria for the classification of CAPS are: (i) ≥3 organs, system, or tissue involvement; (ii) the onset of symptoms occur simultaneously or within a week; (iii) small vessel occlusion, which is confirmed via histological examination; and (iv) aPL positive (LA or aCL) [72]. Confirmation of ‘definite CAPS’ requires all 4 criteria to be fulfilled [72], but this may pose a problem in an acute setting given a considerable overlap with other conditions, such as thrombotic thrombocytopenic purpura/haemolytic uraemic syndrome and other thrombotic microangiopathies. The pathogenesis of CAPS is still not well-understood. The Euro-Phospholipid Project Group reported an incidence of 1% in 10 years whilst 50% of CAPS patients developed it during their first clinical presentation of APS [2,73]. A higher mortality rate was attributed to the presence of SLE [74]. Infections, surgery, and malignancy are the common triggering factors [73]. For patients with thrombotic APS, withdrawal or subtherapeutic level of warfarin has been reported [75,76].

Immediate treatment aims at controlling the thrombotic events and suppressing the overwhelming cytokine storm. Prompt initiation of empirical treatment is crucial given a mortality rate of 35–55% [2,73]. Although there is no randomised trial given the nature of the condition and studies are of limited quality, international guidelines have suggested triple-therapy, which is a combination of steroid, heparin, and plasma exchange or intravenous immunoglobulin [42,77]. A meta-analysis of 357 patients with CAPS showed a reduction in mortality rate among those receiving this combination therapy as compared to those receiving other treatments (OR 0.5; 95% CI: 0.3–1.0) [77].

In our centre, probable or definite CAPS patients are nursed in the intensive care unit with multidisciplinary team management. Anticoagulation with a therapeutic dose of LMWH is commenced as soon as possible. In patients presenting with thrombocytopenia, or thrombocytopenia ensues soon after commencing heparin, we recommend switching to argatroban. Therapeutic plasma exchange is performed every other day until improvement in clinical response is observed. Prednisolone is given at 30 mg daily and reduced by 5 mg every two weeks. The possible triggering factor is treated accordingly at the same time. For long-term management, once the patient’s condition has improved and become stable, anticoagulation therapy is changed to indefinite VKA treatment with a target INR from 3.0–4.0.

## 4. Conclusions

Distinct treatment challenges, approaches, and aims revolve around the holistic management of thrombotic APS. Although the current mainstay of therapy is anticoagulation with VKA, more evidence-based studies are required to delineate which patients may benefit from DOACs, especially those non-high risk APS patients.

## Figures and Tables

**Table 1:** Lupus anticoagulant tests and the main characteristics.

Investigation	Characteristics
aPTT	Automated analysers utilising photo-optical clot detection with micronized silica or ellagic acid as an activator Kaolin is rarely used in automated analysers as its opacity renders clot detection difficult Activates factor XII Is influenced by DOACs and VKAs
Dilute aPTT	Silica activator and a low concentration of phospholipid types that are LA-responsive The confirmatory test involves addition of concentrated platelet-derived phospholipid Is influenced by DOACs and VKAs
dRVVT	RVV directly activates factor X Test is not affected by factors VIII, IX, XI, and XII (intrinsic pathway) Is influenced by DOACs and VKAs
Kaolin clotting test	aPTT test, Kaolin as the activator Without any added phospholipid Phospholipid surface using residual cell membrane fragments and plasma lipids Is influenced by DOACs and VKAs
Silica	aPTT test, Silica as the activator Initiate coagulation via activation of factor XII Contains a low dose of phospholipid Is influenced by DOACs & VKAs
Staclot LA assay system	Using hexagonal (II) phase phosphatidylethanolamine for neutralisation of LA inhibition rather than lamellar phase phospholipid Is influenced by DOACs & VKAs
TSVT	Taipan snake venom directly activates prothrombin Involves dilution of a reference phospholipid preparation Repeated using a platelet neutralisation procedure—substitute PL with washed platelets Is not influenced by VKA as the prothrombin activator in the Taipan venom activates the des-carboxyprothrombin present in the plasma Is influenced by dabigatran as Taipan venom directly activates prothrombin

Abbreviations: aPTT: activated partial thromboplastin time; DOACs: direct oral anticoagulants; dRVVT: dilute Russell viper venom test; LA: lupus anticoagulant; PL: phospholipid; RVV: Russell viper venom; TSVT: Taipan snake venom test.

**Table 2:** Summary of anticoagulants in thrombotic APS.

Site of Thrombosis	aPL Positivity	Warfarin	DOACs
Venous	Single	First choice INR target 2–3	Can be considered *
	Double	First choice INR target 2–3	Can be considered *
	Triple	First choice INR target 2–3	Contraindicated
Arterial	Any	First choice INR target 3–4	Contraindicated

* Patients who have been taking a DOAC may continue or switch to a VKA following a strict discussion and consideration based on their clinical history, treatment adherence, and previous experience. For patients who are not keen to switch, it is recommended to continue DOAC over no anticoagulation (based on the Addendum to British Society for Haematology Guidelines 2020) [43].

## Data Availability

Not applicable.
